# Supplementary material for: Whole-exome sequencing in UK Biobank reveals rare genetic architecture for depression
Source: Nat Commun. 2024 Feb 26;15:1755. doi: 10.1038/s41467-024-45774-2 (PMC10897433; doi:10.1038/s41467-024-45774-2)
Supplement: Supplementary file 1 — Supplementary Information [file 41467_2024_45774_MOESM1_ESM.pdf]

Supplementary materials for

Whole-exome sequencing in UK Biobank reveals rare genetic architecture for depression

Ruoyu Tian, Tian Ge, Hyeokmoon Kweon, Daniel B. Rocha, Max Lam, Jimmy Z. Liu, Kritika Singh, Biogen Biobank Team, Daniel F. Levey, Joel Gelernter, Murray B. Stein, Ellen A. Tsai, Hailiang Huang, Christopher F. Chabris, Todd Lencz, Heiko Runz, Chia-Yen Chen

Correspondence to Chia-Yen Chen (chiayenc@gmail.com) or Heiko Runz (heiko.runz@gmail.com)

Supplementary figures **2**

- Supplementary Figure 1. The exome-wide association of rare variants with seven depression definitions in samples of European ancestry. 2
- Supplementary Figure 2. Down-sampled exome-wide association of rare variants with seven depression definitions in samples of European ancestry. 3
- Supplementary Figure 3. The association of rare variants with seven depression definitions in samples of African ancestry. 5
- Supplementary Figure 4. The association of rare variants with seven depression definitions in samples of South Asian ancestry. 6
- Supplementary Figure 5. Sex-stratified association of rare coding variant burden with EHR-defined depression. 7
- Supplementary Figure 6. Prevalence of depression for PRS percentile in PTV or damaging missense variant carriers and noncarriers. 8
- Supplementary Figure 7. The effects of rare variants in genes with brain specific and non-brain specific expression on EHR-defined depression. 9
- Supplementary Figure 8. Manhattan plot and Q-Q plot of risk gene discovery. 11

Biogen Biobank team **12**

References **13**

Supplementary figures

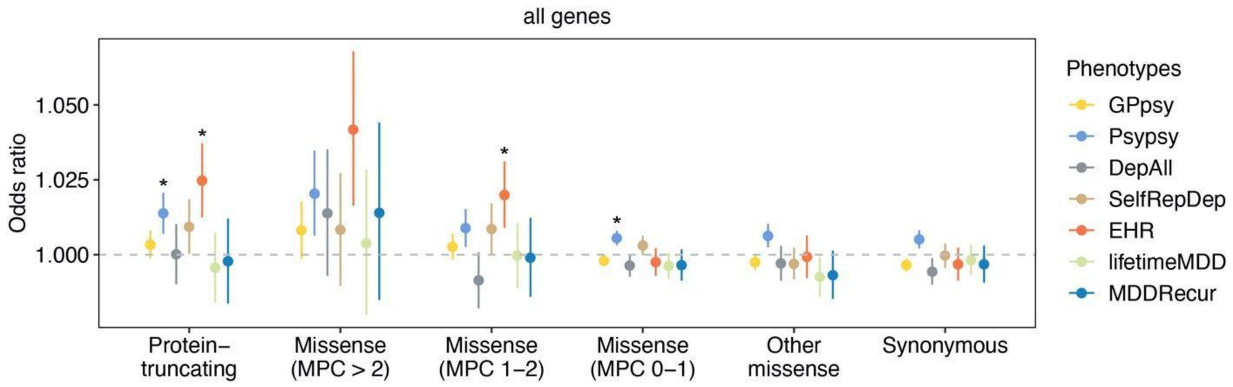

Supplementary Figure 1. The exome-wide association of rare variants with seven depression definitions in samples of European ancestry.

The sample size for each depression definition are as follows: GPpsy: N_{cases} = 111,712, $N_{controls}$ = 206,617; Psypsy: N_{cases} = 36,556, $N_{controls}$ = 282,452; DepAll: N_{cases} = 20,547, $N_{controls}$ = 55,746; SelfRepDep: N_{cases} = 20,120, $N_{controls}$ = 226,578; EHR: N_{cases} = 10,449, $N_{controls}$ = 246,719; lifetimeMD: N_{cases} = 15,580, $N_{controls}$ = 43,104; MDDRecur: N_{cases} = 9,462, $N_{controls}$ = 43,104. Y-axis is the odds ratio (OR) of the association between rare variant burden and depression risk. Rare variants were grouped by functional impact from most to least severe: protein-truncating, missense (MPC > 2, 2 ≥ MPC > 1, 1 ≥ MPC > 0), other missense (missense variants without MPC score annotation) and synonymous variants. The gray dashed line represents the null (OR = 1). Each point shows the point estimate of OR from logistic regression. Bars show 95% confidence intervals (CI). *Bonferroni-adjusted significant $P < 4.20\times10^{-4} = 0.05/119$ (two-sided Wald test; Supplementary Data 3).

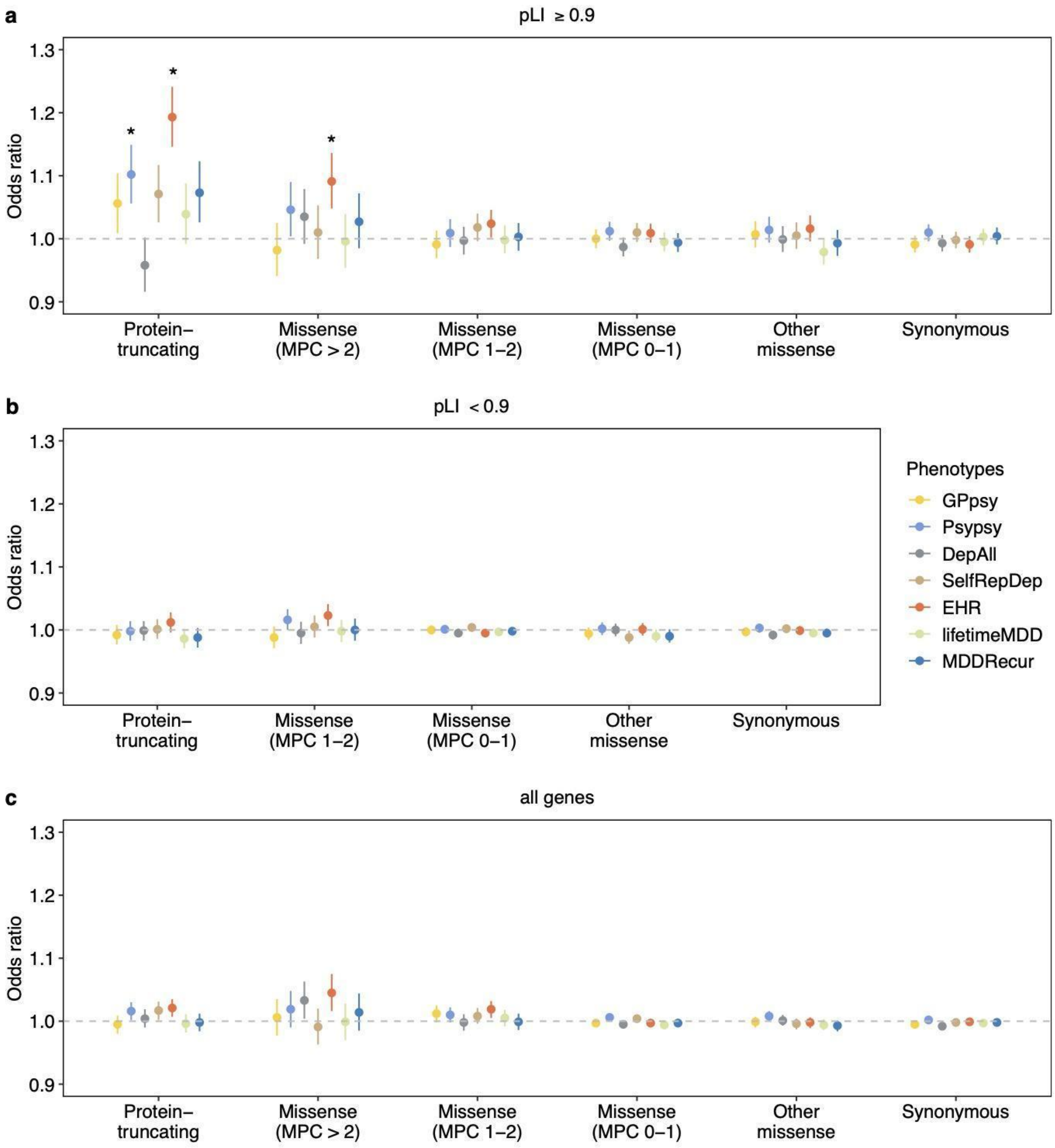

Supplementary Figure 2. Down-sampled exome-wide association of rare variants with seven depression definitions in samples of European ancestry.

In each definition, samples of European ancestry were downsampled to effective sample size equal to 31,035, with the original case prevalence. Y-axis is the odds ratio (OR) of the

association between rare variant burden and depression risk. Protein-coding genes were stratified by pLI into (a) pLI ≥ 0.9, (b) pLI < 0.9 and (c) without stratification (all genes). Rare variants were grouped by functional impact from most to least severe: protein-truncating, missense (MPC > 2, 2 ≥ MPC > 1, 1 ≥ MPC > 0), other missense (missense variants without MPC score annotation) and synonymous variants. The gray dashed line represents the null (OR = 1). Each point shows the point estimate of OR from logistic regression. Bars show 95% confidence intervals (CI). *Bonferroni-adjusted significant $P < 4.20\times10^{-4} = 0.05/119$ (two-sided Wald test; Supplementary Data 5).

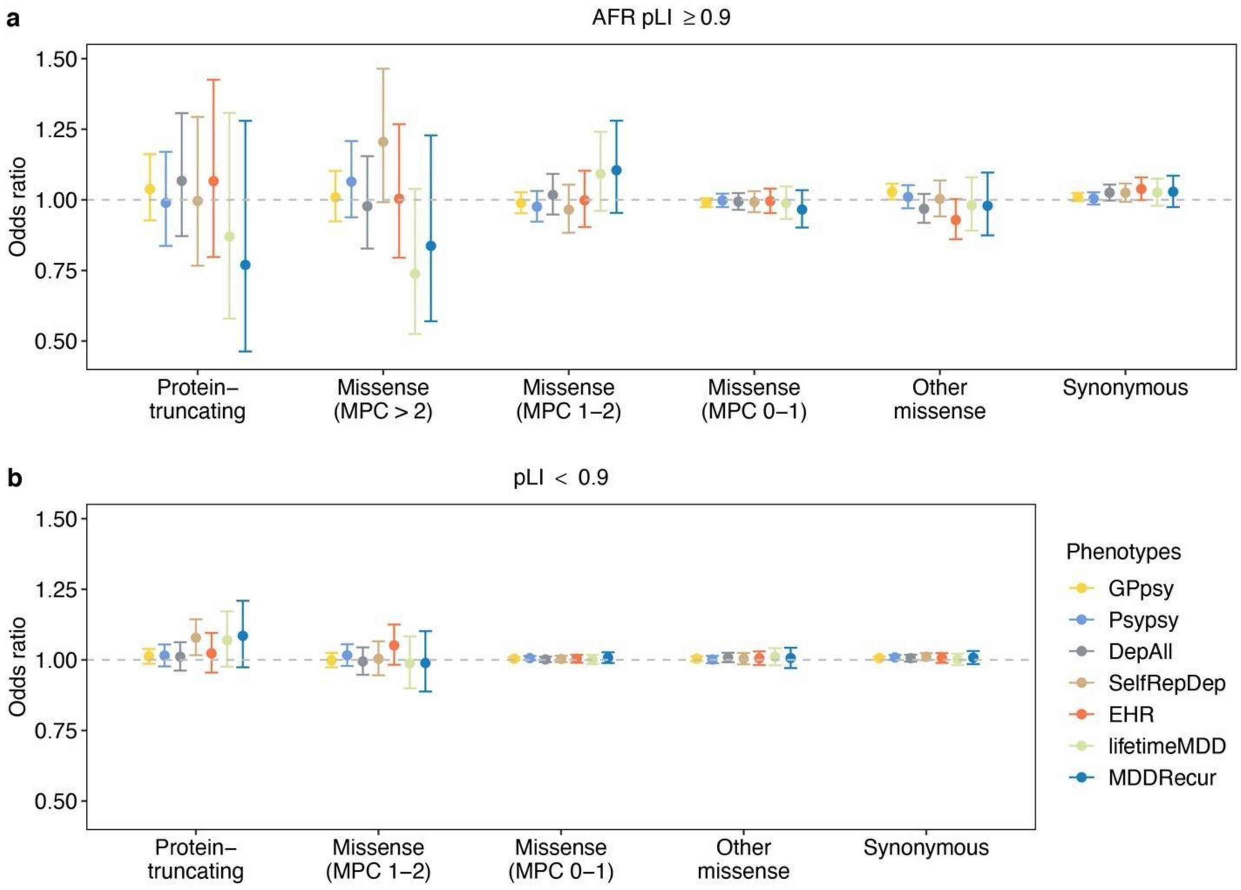

Supplementary Figure 3. The association of rare variants with seven depression definitions in samples of African ancestry.

The sample size for each depression definition are as follows: GPpsy: N_{cases} = 1,457, $N_{controls}$ = 4,668; Psypsy: N_{cases} = 544, $N_{controls}$ = 5,623; DepAll: N_{cases} = 393, $N_{controls}$ = 1,898; SelfRepDep: N_{cases} = 208, $N_{controls}$ = 4,518; EHR: N_{cases} = 155, $N_{controls}$ = 4,758; lifetimeMD: N_{cases} = 129, $N_{controls}$ = 355; MDDRecur: N_{cases} = 87, $N_{controls}$ = 355. Y-axis is the odds ratio (OR) of the association between rare variant burden and depression risk. Protein-coding genes were stratified by pLI, into (a) pLI ≥ 0.9 and (b) pLI < 0.9. Rare variants were grouped by functional impact from most to least severe: protein-truncating, missense (MPC > 2, 2 ≥ MPC > 1, 1 ≥ MPC > 0), other missense (missense variants without MPC score annotation) and synonymous variants.

Missense variants on genes (pLI < 0.9) were only annotated into $2 \geq \text{MPC} > 1$ or $1 \geq \text{MPC} > 0$. The gray dashed line represents the null (OR = 1). Each point shows the point estimate of OR from logistic regression. Bars show 95% confidence intervals (CI). Bonferroni-adjusted significance threshold is $P < 6.49\times10^{-4} = 0.05/77$ (two-sided Wald test; Supplementary Data 6).

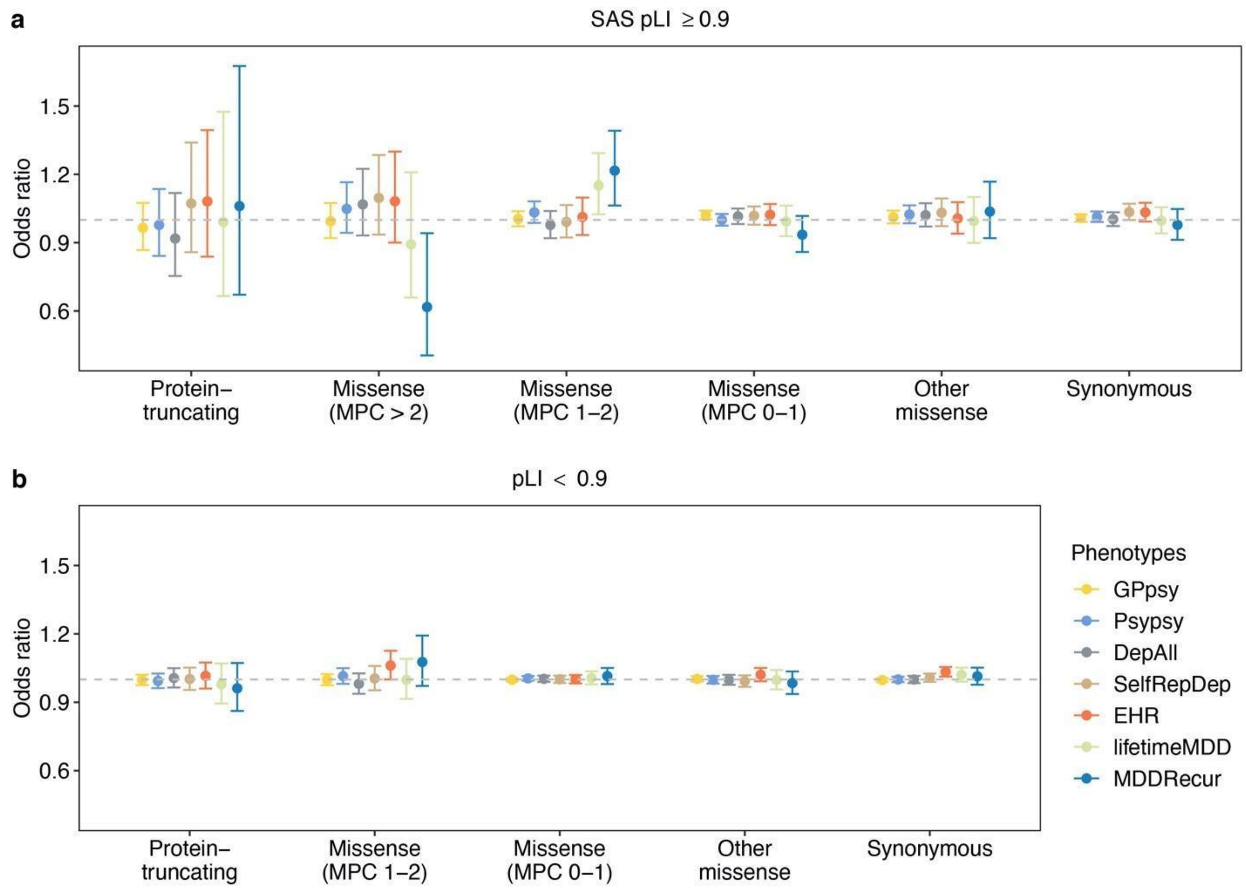

Supplementary Figure 4. The association of rare variants with seven depression definitions in samples of South Asian ancestry.

The sample size for each depression definition are as follows: GPpsy: N_{cases} = 1,534, $N_{controls}$ = 5,185; Psypsy: N_{cases} = 661, $N_{controls}$ = 6,074; DepAll: N_{cases} = 436, $N_{controls}$ = 2,239; SelfRepDep: N_{cases} = 269, $N_{controls}$ = 5,171; EHR: N_{cases} = 196, $N_{controls}$ = 5,453; lifetimeMD: N_{cases} = 140, $N_{controls}$ = 343; MDDRecur: N_{cases} = 89, $N_{controls}$ = 343. Y-axis is the odds ratio (OR) of the association between rare variant burden and depression risk. Protein-coding genes were stratified by pLI, into (a) pLI ≥ 0.9 and (b) pLI < 0.9. Rare variants were grouped by functional impact from most to least severe: protein-truncating, missense (MPC > 2, 2 ≥ MPC > 1, 1 ≥ MPC > 0),

other missense (missense variants without MPC score annotation) and synonymous variants. Missense variants on genes (pLI < 0.9) were only annotated into $2 \geq MPC > 1$ or $1 \geq MPC > 0$. The gray dashed line represents the null (OR = 1). Each point shows the point estimate of OR from logistic regression. Bars show 95% confidence intervals (CI). Bonferroni-adjusted significance threshold is $P < 6.49 \times 10^{-4} = 0.05/77$ (two-sided Wald test; Supplementary Data 6).

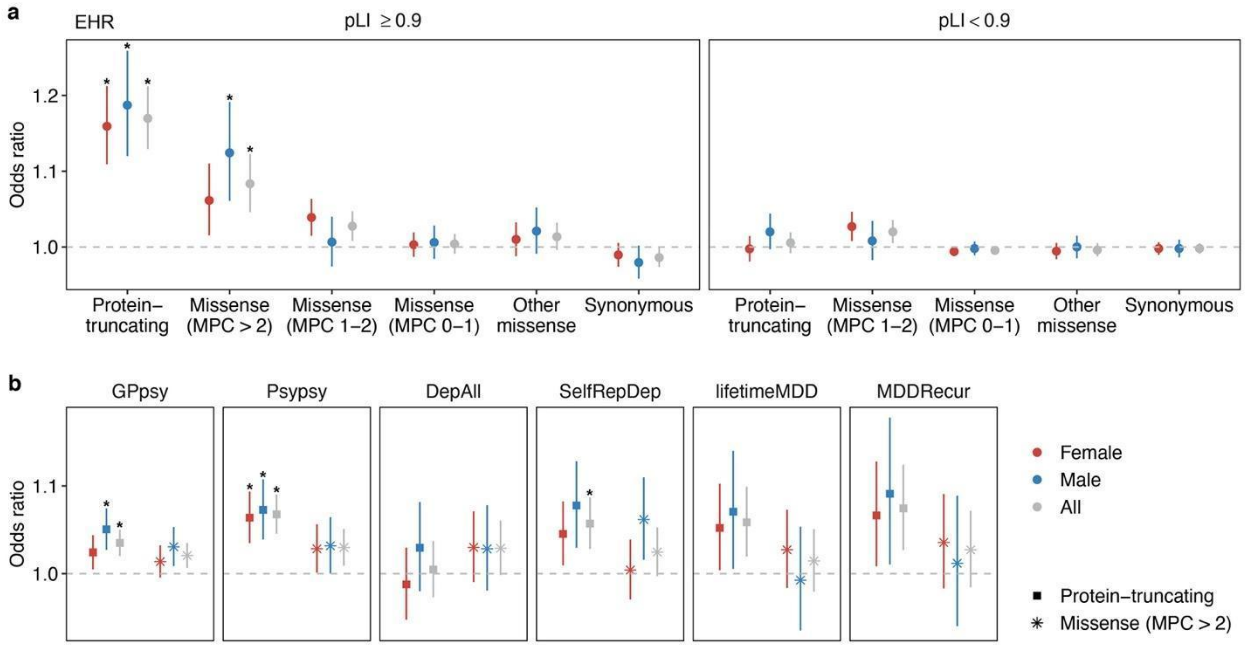

Supplementary Figure 5. Sex-stratified association of rare coding variant burden with EHR-defined depression.

The sample size for each depression definition are as follows: GPpsy: N_{cases} = 111,712, $N_{controls}$ = 206,617; Psypsy: N_{cases} = 36,556, $N_{controls}$ = 282,452; DepAll: N_{cases} = 20,547, $N_{controls}$ = 55,746; SelfRepDep: N_{cases} = 20,120, $N_{controls}$ = 226,578; EHR: N_{cases} = 10,449, $N_{controls}$ = 246,719; lifetimeMD: N_{cases} = 15,580, $N_{controls}$ = 43,104; MDDRecur: N_{cases} = 9,462, $N_{controls}$ = 43,104. Y-axis on each panel is the odds ratio (OR) of the association between rare variant burden and depression risk. (**a**) The effect of 11 groups of rare variants on EHR-defined depression risk in female, male and all subjects. (b) The effect of PTV and damaging missense variants on genes (pLI ≥ 0.9) on GPpsy, Psypsy, DepAll, SelfRepDep, lifetimeMDD and MDDRecur in female, male and all subjects. The gray dashed line represents the null (OR = 1). Each point shows the OR from logistic regression. Bars show 95% confidence intervals (CI) of the point estimate.

*Bonferroni-adjusted significant association for $P < 6.5\times10^{-4} = 0.05/33$ (two-sided Wald test; Supplementary Data 7).

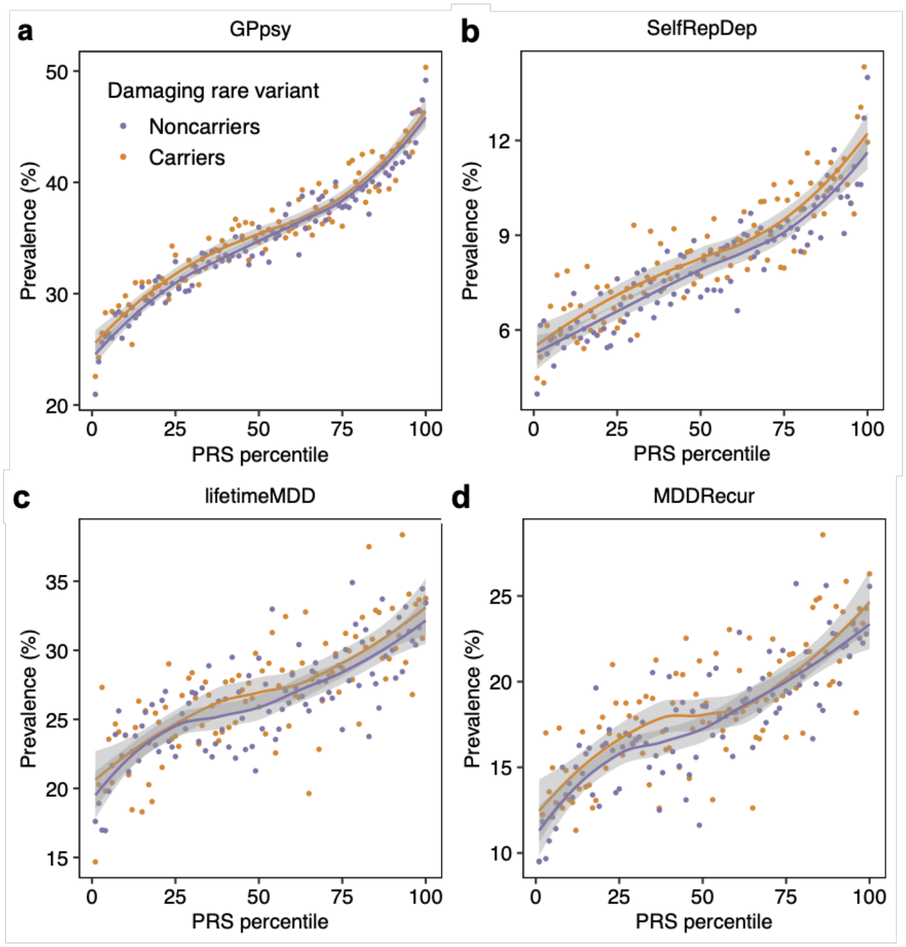

Supplementary Figure 6. Prevalence of depression for PRS percentile in PTV or damaging missense variant carriers and noncarriers.

The prevalence of (a) GPpsy, (b) SelfRepDep, (c) lifetimeMDD, and (d) MDDRecur-defined depression against PRS percentile, stratified by exome-wide PTV or damaging missense variant carrier status. The lines represent the locally fitted regression line by loess regression and gray shading corresponds to the 95% confidence interval of the fitted regression. The sample size for each depression definition are as follows: GPpsy: N_{cases} = 111,712, $N_{controls}$ = 206,617; SelfRepDep: N_{cases} = 20,120, $N_{controls}$ = 226,578; lifetimeMD: N_{cases} = 15,580, $N_{controls}$ = 43,104; MDDRecur: N_{cases} = 9,462, $N_{controls}$ = 43,104.

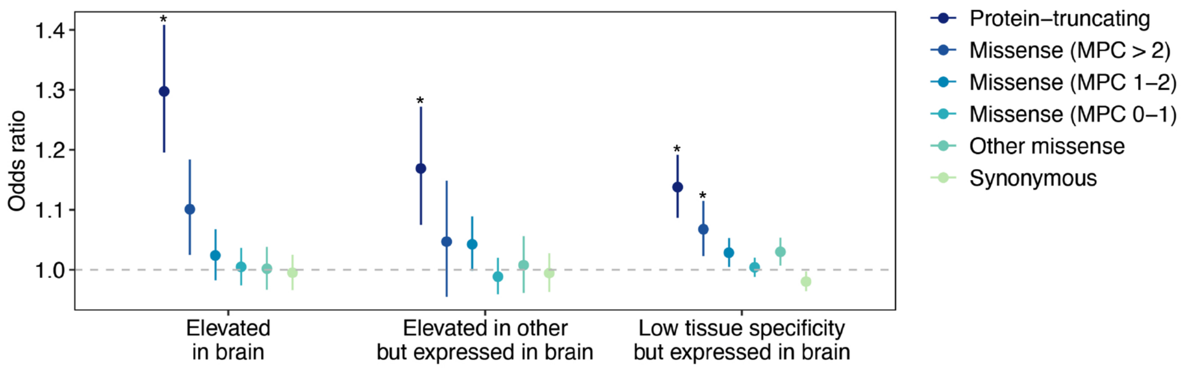

Supplementary Figure 7. The effects of rare variants in genes with brain specific and non-brain specific expression on EHR-defined depression.

We aggregated rare variants of each type (PTV, missense and synonymous) on three human brain atlas gene sets[2], genes with expression elevated in brain (2,587 genes), expression elevated in other tissues but also expressed in brain (5,298 genes) and no tissue specificity but expressed in the brain (8,342 genes). Y-axis is the odds ratio (OR) of the association between rare variant burden for the three gene sets with depression risk. The gray dashed line represents the null (OR = 1). Each point shows the OR from logistic regression. Bars show 95% confidence intervals of OR. *Bonferroni-adjusted significance associations for $P < 3.0\times10^{-3} = 0.05/18$ (two-sided Wald test; Supplementary Data 16). The sample size (sample of European ancestry) for each depression definition are as follows: GPpsy: N_{cases} = 111,712, $N_{controls}$ = 206,617; Psypsy: N_{cases} = 36,556, $N_{controls}$ = 282,452; DepAll: N_{cases} = 20,547, $N_{controls}$ = 55,746; SelfRepDep: N_{cases} = 20,120, $N_{controls}$ = 226,578; EHR: N_{cases} = 10,449, $N_{controls}$ = 246,719; lifetimeMD: N_{cases} = 15,580, $N_{controls}$ = 43,104; MDDRecur: N_{cases} = 9,462, $N_{controls}$ = 43,104.

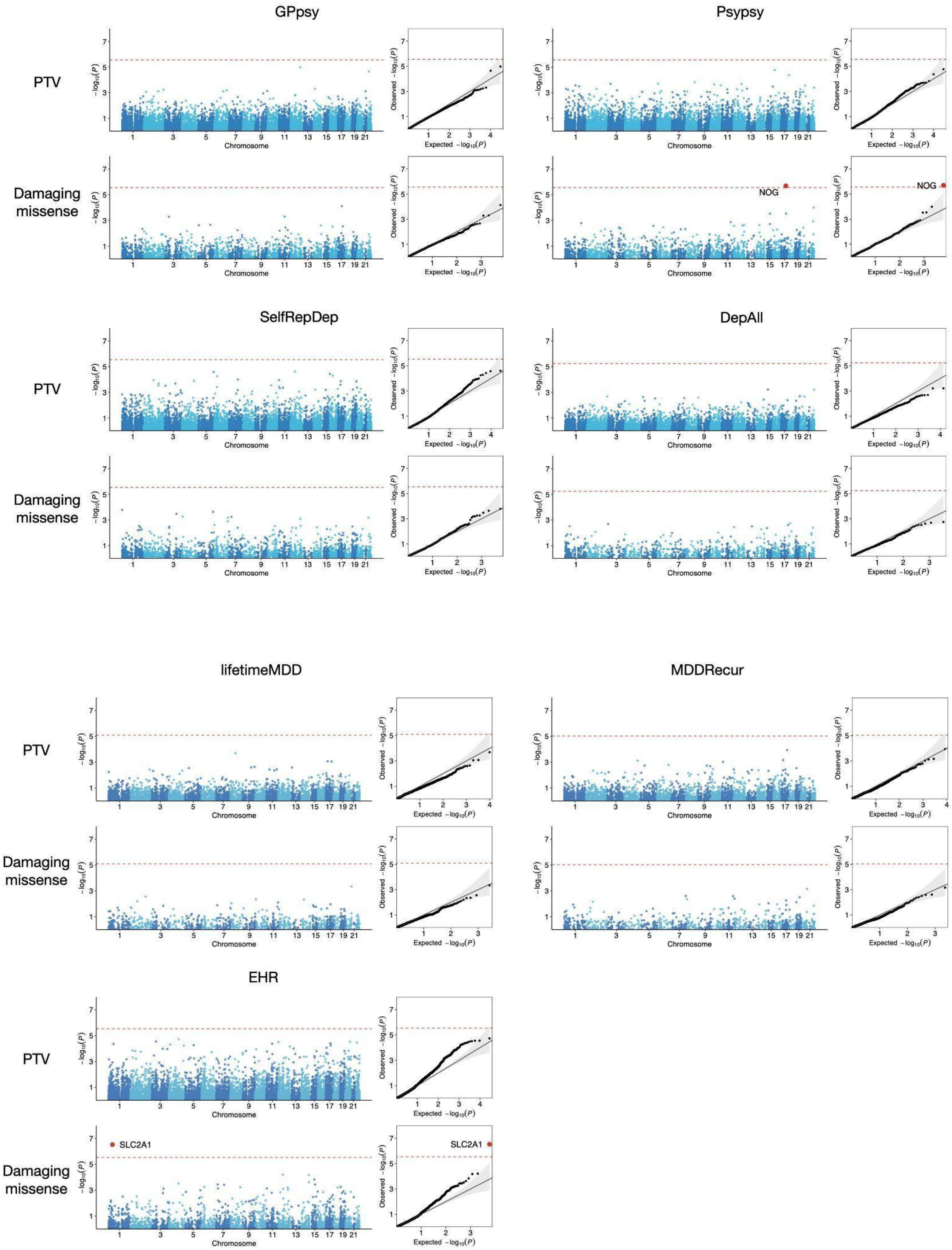
GPpsy
Psypsy
SelfRepDep
DepAll
lifetimeMDD
MDDRecur
EHR
PTV
Damaging missense
NOG
SLC2A1
Chromosome
$-\log_{10}(P)$
Observed $-\log_{10}(P)$
Expected $-\log_{10}(P)$

Supplementary Figure 8. Manhattan plot and Q-Q plot of risk gene discovery.

Manhattan plots of the $-\log_{10} P$ of the association of gene-based PTV and damaging missense variant burden with seven depression definitions. Each dot represents a gene and its genomic location is plotted on the x-axis. Q-Q plots of the observed $-\log_{10} P$ of the association of gene-based PTV and damaging missense variant burden with depression against expected $-\log_{10} P$ under the null (two-sided Wald test). The red dashed line is the per phenotype Bonferroni significant threshold and the red dot represents a significant gene (Supplementary Data 18). The sample size (sample of European ancestry) for each depression definition are as follows: GPpsy: N_{cases} = 111,712, $N_{controls}$ = 206,617; Psypsy: N_{cases} = 36,556, $N_{controls}$ = 282,452; DepAll: N_{cases} = 20,547, $N_{controls}$ = 55,746; SelfRepDep: N_{cases} = 20,120, $N_{controls}$ = 226,578; EHR: N_{cases} = 10,449, $N_{controls}$ = 246,719; lifetimeMD: N_{cases} = 15,580, $N_{controls}$ = 43,104; MDDRecur: N_{cases} = 9,462, $N_{controls}$ = 43,104.

Biogen Biobank team

Steering team: Ellen Tsai, Sally John, Heiko Runz

Data management team: Eric Marshall, Mehool Patel, Saranya Duraisamy

Extended Scientific team: Dennis Baird, Danai Chasioti, Chia-Yen Chen, Susan Eaton, Jake Gagnon, Feng Gao, Cynthia Gubbels, Yunfeng Huang, Stephanie Loomis, Helen McLaughlin, Adele Mitchell, Coro Paisan-Ruiz, Benjamin Sun

References

1. Levey, D. F. *et al.* Bi-ancestral depression GWAS in the Million Veteran Program and meta-analysis in >1.2 million individuals highlight new therapeutic directions. *Nat. Neurosci.* **24**, 954–963 (2021).
2. Sjöstedt, E. *et al.* An atlas of the protein-coding genes in the human, pig, and mouse brain. *Science* **367**, (2020).
