# Supplementary material for: Whole-exome sequencing in UK Biobank reveals rare genetic architecture for depression
Source: Nat Commun. 2024 Feb 26;15:1755. doi: 10.1038/s41467-024-45774-2 (PMC10897433; doi:10.1038/s41467-024-45774-2)
Supplement: Supplementary file 3 — Description of Additional Supplementary Files [file 41467_2024_45774_MOESM3_ESM.pdf]

Description of Additional Supplementary Files

File Name: Supplementary Data 1
Description: Case-Control sample sizes of depression definitions cross populations. Samples sizes in subjects of (a) European, (b) African and (c) South Asian ancestries

File Name: Supplementary Data 2
Description: Statistics of rare variants in cases and controls in EUR samples, including mean, standard deviation, minimum, maximum and median number of (a) PTV, (b) MPC>2, (c) 2≥MPC>1, (d) 1≥MPC>0, (e) other missense variants without MPC annotation and (f) synonymous variants.

File Name: Supplementary Data 3
Description: Exome-wide burden test statistics in EUR samples (P-values are based on two-sided Wald tests).

File Name: Supplementary Data 4
Description: Exome-wide burden test statistics in EUR samples with assessment center as additional covariates (P-values are based on two-sided Wald tests).

File Name: Supplementary Data 5
Description: Exome-wide burden test statistics in EUR samples with down-sampling to effective sample size of 31,035 (P-values are based on two-sided Wald tests).

File Name: Supplementary Data 6
Description: Exome-wide burden test statistics in (a) AFR and (b) SAS samples (P-values are based on two-sided Wald tests).

File Name: Supplementary Data 7
Description: Sex-stratified exome-wide burden test statistics. (a) Test statistics for EHR across all types of rare variants (P-values are based on two-sided Wald tests); (b) Test statistics for GPpsy, Psypsy, DepAll, SelfRepDep, lifetimeMDD and MDDRecur on PTV and damaging missense variant burden of LoF intolerant genes (P-values are based on two-sided Wald tests).

File Name: Supplementary Data 8
Description: Sex-stratified two-sided Poisson exact test in (a) cases and in (b) controls.

File Name: Supplementary Data 9
Description: Genetic correlation based on rare PTVs, missense variants, and common variants between seven depression phenotypes (P-values are based on two-sided Wald tests).

File Name: Supplementary Data 10
Description: Sample sizes, number of significant genomic risk loci, heritability, LD score intercept and mean chi-squared of depression GWAS used in the meta-analysis.

File Name: Supplementary Data 11
Description: Genetic predcition analyses. (a) Univariate estimates of pseudo R2, R2 on the liability scale of PRS, PTV and damaging missense variant (MPC > 2). (b) P-value (two-sided Wald test) for PRS by rare variant carrier status interaction. (c) Comparison of genetic prediction performance between PRS + rare variant burden model vs. PRS only model. (d) Sample size for genetic prediction analysis by depressoin definition

File Name: Supplementary Data 12
Description: Psychiatric and neurodevelopmental disease associated genes from previous GWAS and exome sequencing studies.

File Name: Supplementary Data 13
Description: Burden test statistics in psychiatric and neurodevelopmental disease associated genes for seven depression definitions (P-values are based on two-sided Wald tests).

File Name: Supplementary Data 14
Description: GO gene sets and burden test statistics for PTV and damaging missense variant. (a) Significant GO gene sets and test statistics for damaging missense variant in EHR-defined depression. (b) Significant GO gene sets and burden test statistics for damaging missense variant for MDDRecur-defined depression. (c) Number of tests for each phenotype and Bonferroni significant threshold per phenotype. (All P-values are based on two-sided Wald tests)

File Name: Supplementary Data 15
Description: The human brain atlas genes. (a) 2,587 genes, expression elevated in brain; (b) 5,298 genes, expression elevated in other tissues but expressed in brain; (c) 8,342 genes, expression was not tissue specific but expressed in brain.

File Name: Supplementary Data 16
Description: Burden test statistics in the human brain atlas gene sets for genes with pLI≥0.9 (P-values are based on two-sided Wald tests).

File Name: Supplementary Data 17
Description: Antidepressants interacted genes, depression risk genes interacted drugs and burden test statistics. (a) FDA approved antidepressants and the category each drug belongs to, including tricyclic antidepressants (TCAs), selective serotonin antidepressants (SSRIs), serotonin and norepinephrine reuptake inhibitors (SNRIs) and other (moclobemide). (b) 207 genes interacted with antidepressants listed in (a). (c) The interaction types, source and Pubmed ID of gene-drug interaction between (a) and (b). (d) The summary statistics of associations between rare variants burden on the interacted genes with depression risk (P-values are based on two-sided Wald tests).

File Name: Supplementary Data 18
Description: Depression genes discovery. (a) Genes identified from gene-based burden test. OR: odds ratio; 95% CI: 95% confidence interval. (b) Number of burden tests for each phenotype and Bonferroni significant threshold per phenotype (c) SLC2A1 summary statistics of depression

definitions. (d) SLC2A1 summary statistics in EHR-defined depression in UKB, PBK, DiscoverEHR and meta-analysis. (All P-values are based on two-sided Wald tests.)
