# Supplementary material for: Whole-exome sequencing in UK Biobank reveals rare genetic architecture for depression
Source: Nat Commun. 2024 Feb 26;15:1755. doi: 10.1038/s41467-024-45774-2 (PMC10897433; doi:10.1038/s41467-024-45774-2)
Supplement: Supplementary file 5 — Reporting Summary [file 41467_2024_45774_MOESM5_ESM.pdf]

nature portfolio

Corresponding author(s): Chia-Yen Chen, Heiko Runz

Last updated by author(s): Nov 2, 2023

Reporting Summary

Nature Portfolio wishes to improve the reproducibility of the work that we publish. This form provides structure for consistency and transparency in reporting. For further information on Nature Portfolio policies, see our Editorial Policies and the Editorial Policy Checklist.

Statistics

For all statistical analyses, confirm that the following items are present in the figure legend, table legend, main text, or Methods section.

n/a	Confirmed	
☐	☒	The exact sample size (*n*) for each experimental group/condition, given as a discrete number and unit of measurement
☐	☒	A statement on whether measurements were taken from distinct samples or whether the same sample was measured repeatedly
☐	☒	The statistical test(s) used AND whether they are one- or two-sided<br>*Only common tests should be described solely by name; describe more complex techniques in the Methods section.*
☐	☒	A description of all covariates tested
☐	☒	A description of any assumptions or corrections, such as tests of normality and adjustment for multiple comparisons
☐	☒	A full description of the statistical parameters including central tendency (e.g. means) or other basic estimates (e.g. regression coefficient) AND variation (e.g. standard deviation) or associated estimates of uncertainty (e.g. confidence intervals)
☐	☒	For null hypothesis testing, the test statistic (e.g. *F*, *t*, *r*) with confidence intervals, effect sizes, degrees of freedom and *P* value noted<br>*Give P values as exact values whenever suitable.*
☒	☐	For Bayesian analysis, information on the choice of priors and Markov chain Monte Carlo settings
☒	☐	For hierarchical and complex designs, identification of the appropriate level for tests and full reporting of outcomes
☐	☒	Estimates of effect sizes (e.g. Cohen's *d*, Pearson's *r*), indicating how they were calculated

Our web collection on statistics for biologists contains articles on many of the points above.

Software and code

Policy information about availability of computer code

Data collection	No specific software were employed in data collection.
Data analysis	Software used for analysis includes R v4.2.1, Python v3, burden heritability regression v0.5.0-alpha (https://github.com/ajaynadig/bhr), Variant Effect Predictor (VEP) v96 (https://useast.ensembl.org/info/docs/tools/vep/index.html), LOFTEE (https://github.com/konradjk/loftee), PRS-CS v1.0.0 (https://github.com/getian107/PRScs), PLINK1.90b (https://www.cog-genomics.org/plink/), PLINK2.00a (https://www.cog-genomics.org/plink/2.0), Hail v0.2 (https://github.com/hail-is/hail), and LD Score regression v1.0.1 (https://github.com/bulik/ldsc). Analysis codes used in this manuscript can be found at https://doi.org/10.5281/zenodo.10511823.

For manuscripts utilizing custom algorithms or software that are central to the research but not yet described in published literature, software must be made available to editors and reviewers. We strongly encourage code deposition in a community repository (e.g. GitHub). See the Nature Portfolio guidelines for submitting code & software for further information.

Data

Policy information about availability of data

All manuscripts must include a data availability statement. This statement should provide the following information, where applicable:

- Accession codes, unique identifiers, or web links for publicly available datasets
- A description of any restrictions on data availability
- For clinical datasets or third party data, please ensure that the statement adheres to our policy

The full gene burden association results from UK Biobank in this study can be found at https://doi.org/10.5281/zenodo.10511823. All phenotypic and genotypic data for the UK Biobank are available to researchers with approved data access from the UK Biobank (https://www.ukbiobank.ac.uk/enable-your-research/register). MGBB data are not publicly available due to privacy and ethical restrictions. Please contact the MGBB for further information on data access (https://www.massgeneralbrigham.org/en/research-and-innovation/participate-in-research/biobank/for-researchers). Please contact the Geisinger DiscovEHR for further information on data access (https://www.geisinger.org/precision-health/mycode/discovehr-project). GWAS summary statistics from FinnGen can be downloaded at https://www.finngen.fi/en/access_results. Meta-analysis of depression by PGC (excluding UK Biobank and 23andme participants) can be downloaded at https://www.med.unc.edu/pgc/download-results/mdd/. Summary statistics of GWAS on samples of European ancestry in Million Veteran Program (MVP) was obtained through MVP Project Proposal MVP200097. pLoF Metrics is available at https://storage.googleapis.com/gcp-public-data--gnomad/release/2.1.1/constraint/gnomad.v2.1.1.lof_metrics.by_gene.txt.bgz. The MPC score is available at ftp://ftp.broadinstitute.org/pub/ExAC_release/release1/regional_missense_constraint/. Human protein atlas data is available at https://www.proteinatlas.org/humanproteome/brain/human+brain. Drug gene interaction database is available at https://www.dgidb.org/.

Research involving human participants, their data, or biological material

Policy information about studies with human participants or human data. See also policy information about sex, gender (identity/presentation), and sexual orientation and race, ethnicity and racism.

Reporting on sex and gender	Sex information was collected by self-report in each biobank. To control for confounding, we adjusted sex as a covariate in all association analyses. We also conducted sex-stratified exome-wide burden analyses in UK biobank.
Reporting on race, ethnicity, or other socially relevant groupings	To adjust for potential population stratification in association analysis, we assigned biobank participants into five major populations, including European (EUR), East Asian (EAS), African (AFR), American (AMR) and South Asian (SAS), based on their genotype data (see Methods for detailed description). Association analyses were conducted within each population. To account for population stratification, we adjusted for top 20 principal components derived from genotype data as covariates in all association analyses for each population separately.
Population characteristics	The UK Biobank is a large prospective population-based study with over half a million participants recruited across the UK. Participants had age between 40 to 69 years at recruitment in 2006-2010 and provided extensive phenotype data, including surveys on baseline characteristics and health outcomes, specific questionnaires and assessments, health records, physical measures and biomarkers. A total of 454,787 whole-exome sequenced UK Biobank participants were included in this study. See http://www.ukbiobank.ac.uk/ for additional UK Biobank participant information.
Recruitment	The UK Biobank recruited about 500,000 participants in the UK, enrolled at ages from 40 to 69 from 2006 to 2010. The MGBB and DiscoverEHR are both hospital-based biobanks where participants volunteer to enter the study.
Ethics oversight	Collection of the UK Biobank (UKB) data was approved by the UKB's Research Ethics Committee. UKB individual-level, phenotypic data and whole exome sequencing data used in the present work were obtained under application ID 26041. The complete whole exome sequencing data used in this study will be publicly available once the embargoes have lifted (expected 2021).<br><br>The Ethics Committee of Mass General Brigham approved the research protocol (2009-P-002312).<br><br>Patients and control subjects in FinnGen provided informed consent for biobank research, based on the Finnish Biobank Act. Alternatively, separate research cohorts, collected prior the Finnish Biobank Act came into effect (in September 2013) and start of FinnGen (August 2017), were collected based on study-specific consents and later transferred to the Finnish biobanks after approval by Fimea, the National Supervisory Authority for Welfare and Health. Recruitment protocols followed the biobank protocols approved by Fimea. The Coordinating Ethics Committee of the Hospital District of Helsinki and Uusimaa (HUS) approved the FinnGen study protocol Nr HUS/990/2017.<br><br>The FinnGen study is approved by Finnish Institute for Health and Welfare (permit numbers: THL/2031/6.02.00/2017, THL/1101/5.05.00/2017, THL/341/6.02.00/2018, THL/2222/6.02.00/2018, THL/283/6.02.00/2019, THL/1721/5.05.00/2019, THL/1524/5.05.00/2020, and THL/2364/14.02/2020), Digital and population data service agency (permit numbers: VRK43431/2017-3, VRK/6909/2018-3, VRK/4415/2019-3), the Social Insurance Institution (permit numbers: KELA 58/522/2017, KELA 131/522/2018, KELA 70/522/2019, KELA 98/522/2019, KELA 138/522/2019, KELA 2/522/2020, KELA 16/522/2020 and Statistics Finland (permit numbers: TK-53-1041-17 and TK-53-90-20).<br><br>The Biobank Access Decisions for FinnGen samples and data utilized in FinnGen Data Freeze 6 include: THL Biobank BB2017_55, BB2017_111, BB2018_19, BB_2018_34, BB_2018_67, BB2018_71, BB2019_7, BB2019_8, BB2019_26, BB2020_1, Finnish Red Cross Blood Service Biobank 7.12.2017, Helsinki Biobank HUS/359/2017, Auria Biobank AB17-5154, Biobank Borealis of Northern Finland_2017_1013, Biobank of Eastern Finland 1186/2018, Finnish Clinical Biobank Tampere MH0004, Central Finland Biobank 1-2017, and Terveystalo Biobank STB 2018001.<br><br>Meta-analysis of depression by PGC (without UK Biobank and 23andme participants) is available at https://

www.med.unc.edu/pgc/download-results/mdd/.

Summary statistics of GWAS on individuals with European ancestry from Million Veteran Program (MVP) cohort was obtained through MVP Project Proposal MVP200097.

Note that full information on the approval of the study protocol must also be provided in the manuscript.

Field-specific reporting

Please select the one below that is the best fit for your research. If you are not sure, read the appropriate sections before making your selection.

☒ Life sciences ☐ Behavioural & social sciences ☐ Ecological, evolutionary & environmental sciences

For a reference copy of the document with all sections, see nature.com/documents/nr-reporting-summary-flat.pdf

Life sciences study design

All studies must disclose on these points even when the disclosure is negative.

Sample size	See Methods for detailed description on sample sizes for UK Biobank, MGBB and DiscoverEHR. Sample size was chosen to maximize the power for gene discovery (i.e. as large as possible) from each biobank and genome-wide association study with relevant depression phenotypes, covariates, whole-exome sequencing and/or genome-wide genotype data.
Data exclusions	See Methods for data quality control details. We excluded individuals whose reported gender differed from genetic sex or who had sex chromosome aneuploidies, individuals withdrawn from the UK Biobank, and individuals with missing phenotypes.
Replication	Replication of depression genetic association findings in UK Biobank was performed in two independent biobanks, MGBB and DiscoverEHR. At current sample sizes, SLC2A1 burden association was not replicated in the Geisinger DiscovEHR cohort or the Mass General Brigham Biobank, although these burden associations showed consistent directions of effect.
Randomization	Randomization is not applicable as this is an observational study.
Blinding	Blinding is not applicable as this is an observational study.

Reporting for specific materials, systems and methods

We require information from authors about some types of materials, experimental systems and methods used in many studies. Here, indicate whether each material, system or method listed is relevant to your study. If you are not sure if a list item applies to your research, read the appropriate section before selecting a response.

Materials & experimental systems

n/a	Involved in the study	
☒	☐	Antibodies
☒	☐	Eukaryotic cell lines
☒	☐	Palaeontology and archaeology
☒	☐	Animals and other organisms
☒	☐	Clinical data
☒	☐	Dual use research of concern
☒	☐	Plants

Methods

n/a	Involved in the study	
☒	☐	ChIP-seq
☒	☐	Flow cytometry
☒	☐	MRI-based neuroimaging

Plants

Seed stocks	*Report on the source of all seed stocks or other plant material used. If applicable, state the seed stock centre and catalogue number. If plant specimens were collected from the field, describe the collection location, date and sampling procedures.*
Novel plant genotypes	*Describe the methods by which all novel plant genotypes were produced. This includes those generated by transgenic approaches, gene editing, chemical/radiation-based mutagenesis and hybridization. For transgenic lines, describe the transformation method, the number of independent lines analyzed and the generation upon which experiments were performed. For gene-edited lines, describe the editor used, the endogenous sequence targeted for editing, the targeting guide RNA sequence (if applicable) and how the editor was applied.*
Authentication	*Describe any authentication procedures for each seed stock used or novel genotype generated. Describe any experiments used to assess the effect of a mutation and, where applicable, how potential secondary effects (e.g. second site T-DNA insertions, mosiacism, off-target gene editing) were examined.*
